# Therapeutic Dilemmas in the Management of Stroke Among Patients With Infective Endocarditis (IE): A Literature Review

**DOI:** 10.7759/cureus.96817

**Published:** 2025-11-14

**Authors:** Sikandar Hayat, Affan Faisal, Hasaan Ans, Ali Raza Rana, Mustehsan Ali

**Affiliations:** 1 General Surgery, Services Hospital Lahore, Lahore, PAK; 2 General Surgery, Chaudhary Muhammad Akram Teaching and Research Hospital, Lahore, PAK; 3 Medicine and Surgery, Mayo Hospital, Lahore, PAK; 4 Internal Medicine, Fatima Memorial Hospital (FMH) College of Medicine and Dentistry, Lahore, PAK; 5 Neurology, Mayo Hospital, Lahore, PAK; 6 Medicine and Surgery, Faisalabad Medical University, Faisalabad, PAK

**Keywords:** antimicrobial therapy, cerebral embolism, echocardiography, infective endocarditis, mechanical thrombectomy, mycotic aneurysm, staphylococcus aureus, stroke

## Abstract

Infective endocarditis (IE) is an infection of the endocardial lining of the heart, with high morbidity and mortality. Neurological complications, mainly stroke, represent the most frequent and devastating extracardiac manifestations. Despite continued advancements in diagnostics and management, the optimal strategies and guidelines to prevent and treat IE-associated stroke remain controversial and scarce.

We conducted a detailed narrative review of recent literature focusing on the epidemiology, risk factors, pathophysiology, diagnostic approaches, and therapeutic strategies for stroke in the context of IE. Evidence was drawn from multinational cohort studies, systematic reviews, guideline statements, and recent updates in imaging, surgical practices, and guidelines. Stroke leads to complications in approximately 15%-20% of IE cases, with regional variation linked to healthcare-associated infections, intravenous drug use, and rheumatic or congenital heart disease. Risk factors include *Staphylococcus aureus* infection, vegetation size >10 mm, hypertension, atrial fibrillation, and cerebral microbleeds. Advanced neuroimaging techniques (MRI, CTA, and MRA) aid in the early detection of ischemic and hemorrhagic events, while updated Duke-ISCVID (International Society of Cardiovascular Infectious Diseases) criteria improve diagnostic accuracy. Antimicrobial therapy reduces embolic risk, but the role of anticoagulants and antiplatelet agents remains controversial. Intravenous thrombolysis is contraindicated due to high hemorrhagic risk, whereas mechanical thrombectomy appears safe and effective only in carefully selected patients. The timing of cardiac surgery requires multidisciplinary decision-making: early surgery may prevent embolism but is associated with increased perioperative neurological complications, particularly in hemorrhagic stroke. Mycotic aneurysms, though uncommon, call for early diagnosis and tailored endovascular or surgical management. IE-related stroke poses complex diagnostic and therapeutic challenges that require multidisciplinary care involving the integration of cardiology, neurology, infectious disease, and surgical expertise. Advances in imaging, early antimicrobial therapy, optimized surgical timing, and cautious use of reperfusion techniques are reshaping management. Ongoing research is needed to refine risk stratification and optimize individualized care.

## Introduction and background

Infective endocarditis (IE) represents a complex intersection of cardiovascular infection and systemic complications, making it one of the most challenging conditions encountered in clinical practice - a challenge faced by many physicians, including neurologists, infectious disease physicians, and emergency physicians. Neurological complications, particularly stroke, remain among the most feared outcomes due to their frequency, morbidity, and impact on treatment decisions and overall prognosis. Recent advances in diagnostic imaging, molecular microbiology, and surgical techniques have improved case detection and management; however, controversies persist regarding antithrombotic therapy (ATT) and the timing of surgery. A comprehensive review of the evolving literature is therefore essential to guide clinicians in balancing infection control, stroke prevention, and individualized therapeutic strategies.

IE is an infection of the endocardial surface of the heart, including valves and intracardiac devices, characterized by the direct invasion of fungi, bacteria, or other microorganisms circulating in the bloodstream. It is mainly a disease caused by bacteria and has a range of manifestations and sequelae. Without early identification and treatment, many intracardiac and far-reaching extracardiac complications can develop. Therefore, a thorough history and physical exam can help diagnose cases and ultimately guide management, limiting mortality and morbidity [1]. The 1994 Modified Duke Criteria were used to diagnose IE, but had their limitations in complex cases. The 2023 Duke-ISCVID (International Society of Cardiovascular Infectious Diseases) Criteria introduced advanced imaging and molecular tools to improve diagnostic accuracy. This highlights the importance of further research to adapt to evolving clinical challenges [2].

## Review

Methodology

This article is a narrative review designed to summarize and synthesize current evidence regarding stroke management in patients with IE. A comprehensive literature search was conducted using PubMed, Scopus, and Google Scholar databases from inception to March 2025. The search strategy combined keywords and Medical Subject Headings (MeSH), such as “infective endocarditis,” “stroke,” “neurological complications,” “mycotic aneurysm,” “antithrombotic therapy,” and “mechanical thrombectomy.” Reference lists of key publications and relevant review articles were also screened to identify additional studies.

Eligible articles included clinical studies (retrospective, prospective, cohort, and observational), meta-analyses, and review papers published in English that discussed the epidemiology, diagnosis, pathophysiology, or management of IE-related stroke (IERS). Case reports and small series were selectively included when they provided novel insights into rare neurological complications. Studies not focused on stroke or neurological manifestations of IE were excluded.

No formal statistical synthesis, meta-analysis, or quantitative pooling was performed. Findings were summarized descriptively to highlight clinical trends, diagnostic advances, and therapeutic dilemmas. Accordingly, statistical parameters (e.g., p-values and confidence intervals) are reported only when explicitly available in the cited literature. As this review is narrative in scope, no formal risk of bias assessment was undertaken.

The subsequent sections are organized to reflect the clinical continuum of IE, from epidemiology and risk factors to diagnostic evaluation, therapeutic strategies, and surgical management, with an emphasis on stroke-related complications.

Cardiac and extracardiac complications of IE

IE has a high risk of serious complications, both intracardiac and extracardiac. These include valvular incompetence, abscesses, heart block, and embolic events (EEs). Right-sided vegetations result in arterial emboli that lead to pulmonary abscesses or focal pulmonary infarctions. On the other hand, left-sided emboli can result in neurologic complications, which make up the most severe and widespread extracardiac complications, notably stroke [3]. 

Another study highlights that neurologic complications can sometimes be overt or subtle (microhemorrhages on MRI) and may even be asymptomatic, making them difficult to detect clinically in patients with severe cardiac failure. Management of patients with these complications of endocarditis, particularly those with mycotic aneurysms or intracerebral hemorrhages (ICHs), can be challenging. These findings understandably raise concern about valve surgery when indicated, due to the risk of hemorrhage associated with perioperative anticoagulation [4].

Epidemiology

Global Epidemiology of IE

Globally, IE has shown a rising incidence over the past three decades. A study that examined temporal trends in IE from 1990 to 2021, sourcing data from the Global Burden of Disease Study (GBD) 2021, showed that the age-standardized incidence rate (ASIR) of IE increased from 9.35 per 100,000 in 1990 to 12.61 per 100,000 in 2021. Despite this increase in incidence, the age-standardized mortality rate (ASMR) has remained stable, and the age-standardized DALY rate (ASDR) has shown a gradual decline, reflecting potential improvements in diagnosis and care. The burden of IE was found to be higher among males and elderly populations, with the greatest increases in incidence and burden occurring in high socio-demographic index (SDI) regions. In contrast, low SDI regions experienced relatively high mortality and disability rates, highlighting disparities in healthcare access and outcomes. These findings emphasize the persistent and shifting global burden of IE and suggest the need for region-specific prevention and management strategies to address its complex epidemiological profile [5].

Another study reported an increase in ASMR from 0.73 to 0.87 per 100,000 person-years over the past 30 years. In 2019, the highest ASMR was observed in high SDI regions, which also experienced the largest rise over three decades. The affected population gradually shifted from younger to older individuals [6].

Stroke as a Major Complication of IE

A large multinational cohort study by the International Collaboration on Endocarditis (ICE), involving 2,781 patients with definitive IE, reported stroke in approximately 17% of cases, making it the most frequent neurological complication. The majority of these strokes had an early onset. *Staphylococcus aureus* infection, mitral valve involvement, and larger vegetations (>10 mm) were significantly associated with increased stroke risk. Most events were ischemic, and stroke presence was linked to greater rates of heart failure and EEs. Despite these complications, patients who experienced stroke underwent early surgery more frequently, and, when managed appropriately, neurologic events did not significantly increase in-hospital mortality. These findings highlight the importance of early detection and management of neurological complications, especially stroke. Significant regional variation in the incidence and presentation of stroke is seen [3].

Geographic Variations in IERS

In North America, the highest rate of neurological complications was observed, with stroke occurring in 21% of cases, largely associated with healthcare-related infections and intravenous drug use. Europe showed an intermediate stroke incidence (~16%) and a mix of community- and healthcare-acquired cases, with both staphylococci and streptococci commonly implicated. In South America, stroke incidence was comparable to that in Europe (~18%), but rheumatic heart disease (RHD) remained a common predisposing factor, particularly among younger individuals. Meanwhile, the Asia-Pacific region had the lowest stroke incidence (11%), with a higher prevalence of underlying congenital heart disease and RHD. Access to cardiac surgery and advanced care was more limited in South America and the Asia-Pacific region, contributing to regional disparities in outcomes. These findings underscore the importance of considering regional epidemiological differences when designing diagnostic, preventive, and treatment strategies [3].

Risk factors for IE-associated stroke

A meta-analysis was conducted to identify risk factors for stroke in patients with IE. Among 35 included studies, *S. aureus* infection and increasing vegetation size were significantly associated with acute ischemic stroke (AIS). Additional risk factors included high-intensity signals on transcranial Doppler, hypertension, atrial fibrillation, and hyperlipidemia. Risk factors for intracranial hemorrhage (ICH) included thrombocytopenia, prior AIS or ICH, cerebral microbleeds (CMBs), and mycotic aneurysms. The findings suggest that both clinical and imaging parameters can help in predicting and thus reducing stroke incidence in IE [7].

Large vegetation size is associated with stroke in patients with IE. Mycotic aneurysms are found at a higher frequency in young patients and are the primary cause of intraparenchymal hemorrhage. CMBs may be related to prosthetic valves and *S. aureus* infection [8]. Hyperlipidemia, hypertension, age, vegetation size (>10 mm), *S. aureus* infection, and early prosthetic valve IE were closely correlated with ischemic stroke [8].

A retrospective study, done in the greater Cincinnati region, pointed out the role of intravenous drug abuse (IVDU) as a risk factor for IERS. The percentage of IERS patients with IVDU increased markedly from 8.3% in 2005 to 44.0% in 2015, indicating a strong temporal association (Table 1) [9].

**Table 1:** Risk factors for stroke in infective endocarditis

Risk Factor/Predictor	Associated Stroke Type or Effect	Reference
*Staphylococcus aureus* infection	Increased risk of acute ischemic stroke	[7,8]
Large vegetation size (>10 mm)	Increased risk of ischemic stroke	[7,8]
Hypertension	Increased risk of ischemic stroke	[7]
Atrial fibrillation	Increased risk of ischemic stroke	[7]
Hyperlipidemia	Increased risk of ischemic stroke	[7]
Advanced age	Increased risk of ischemic stroke	[8]
Early prosthetic-valve infective endocarditis (IE)	Increased risk of ischemic stroke	[8]
Thrombocytopenia	Increased risk of intracranial hemorrhage	[7]
Prior ischemic or hemorrhagic stroke	Higher risk of intracranial hemorrhage	[7]
Cerebral microbleeds (CMB)	Higher risk of intracranial hemorrhage	[7,8]
Mycotic aneurysms	Major cause of intracranial hemorrhage	[7,8]
Intravenous drug use (IVDU)	Increased incidence of IE-related stroke	[9]

Pathophysiology of IERS

EEs with underlying IE also show unusual manifestations, such as the potential for hemorrhagic conversion of stroke. Cerebral complications in IE are mainly due to embolization. Predicting which patients will experience EEs is challenging, and the benefit of cardiac surgery for prevention, in the absence of heart failure or uncontrolled infection, remains uncertain due to limited retrospective evidence [10].

In IE, the hallmark lesion is the vegetation, an infected mass attached to the endocardium or a cardiac implant. The process often begins with endothelial damage, which exposes the subendothelial extracellular matrix, activates platelets, and leads to the formation of a sterile fibrin-platelet clot. Bloodborne microorganisms then adhere to this clot, promoting vegetation growth. The eventual embolization of these vegetations can cause cerebral artery blockage and ischemic stroke [11].

Mycotic aneurysms 

Mycotic aneurysms arise when septic emboli lodge in the vasa vasorum or arterial lumen, triggering inflammatory destruction of the vessel wall and aneurysm formation in cerebral arteries. A large cohort study found that mycotic aneurysms occur in 1.9% of IE cases, with almost 50% located intracranially. Intracranial rupture of mycotic aneurysms is common and associated with high mortality. Early intervention in unruptured mycotic aneurysms may reduce rupture risk and improve outcomes [12]. Mycotic aneurysm rupture can cause intracranial, intraventricular, or subarachnoid hemorrhage, and these aneurysms represent roughly 5% of neurological complications in patients with left-sided infective endocarditis (LSIE) [13].

Hemorrhagic stroke and CMBs in IE

Hemorrhagic strokes account for almost 30% of cerebrovascular complications associated with IE and result from hemorrhagic transformation of infarcts, ICH, or rupture of mycotic aneurysms [14]. In a retrospective study that evaluated patients with active IE who underwent brain MRI to assess the presence of CMBs, CMBs were identified in 54% of patients and were associated with older age, prior antiplatelet therapy, *Staphylococcus* infection, and prosthetic valve endocarditis. Despite these associations, there were no significant differences between patients with and without CMBs in terms of postoperative stroke incidence, in-hospital mortality, or one-year major adverse events [15].

Stroke as the initial presentation of IE

Stroke may present before the diagnosis of IE is made. A retrospective study of 34 IE-associated stroke cases revealed that most patients (26/34) were diagnosed with IE after presenting with stroke, with a median diagnostic delay of eight days. Diagnostic delay did not affect survival but highlights how stroke frequently precedes IE recognition, especially in culture-negative cases [16].

Endocarditis prevalence in patients admitted for a primary diagnosis of aortic stenosis (AS) is low, but this etiology leads to a poor outcome. Some laboratory, clinical-epidemiological, and echocardiographic parameters may help the physician to recognize this condition early and, consequently, to promptly start antibiotic therapy [17].

A 75-year-old patient presented with a stroke linked to subclinical IE. The transesophageal echocardiography (TEE) revealed vegetations on all aortic valve cusps, and three afebrile blood cultures grew *Abiotrophia defectiva*. Despite the absence of typical infection signs, the severe valve involvement required bioprosthetic replacement [18]. This clinical case emphasizes the importance of investigating the infective origin of endocarditis, even in the absence of clinical or laboratory evidence. 

A 31-year-old woman presented with right-sided weakness and was found to have subarachnoid and intraparenchymal hemorrhages. Imaging revealed a left middle cerebral artery occlusion and a saccular aneurysm, later confirmed to be a partially thrombosed pseudoaneurysm. Further evaluation of the patient identified mitral valve vegetation, leading to a diagnosis of IE [19].

Although rare, a hemorrhagic stroke can be the initial presentation of IE, so its recognition is vital to avoid delays in diagnosis and to guide urgent management.

Diagnostic imaging in IE and stroke

In 2023, the Duke criteria were updated by ISCVID to include broader microbiologic and imaging modalities. Under the 2023 Duke-ISCVID criteria, typical IE pathogens recovered from two or more separate blood culture sets constitute a major diagnostic criterion, while less typical organisms must be isolated from three or more sets. The new criteria remove rigid timing requirements for cultures and formally incorporate molecular diagnostics (e.g., PCR and metagenomic sequencing) and non-invasive imaging modalities (such as 18F-FDG PET/CT and cardiac CT). Importantly, intraoperative inspection during surgery is now a recognized major endocardial criterion, and predisposition criteria have been expanded to include transcatheter valves and cardiac devices [2,14].

**Figure 1:**
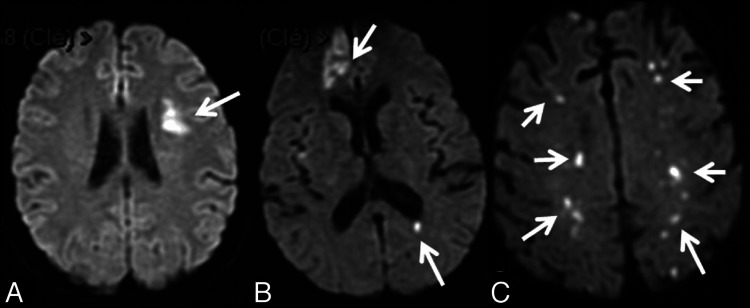
DWI MRI showing acute ischemic lesions in three different patients (A) Single territorial infarct; (B) Territorial plus small cortical/subcortical infarcts; (C) Multiple small watershed ischemic lesions (arrows). Image credit: Hess et al. [14] MRI, magnetic resonance imaging; DWI, diffusion-weighted imaging

MRI plays a crucial role in the early detection of silent neurological complications in IE. As shown in Figure 1, diffusion-weighted MRI frequently demonstrates multiple small acute ischemic lesions in patients with IE [14].

In a prospective study of asymptomatic IE patients, MRI identified brain abnormalities in over 70% of cases, most commonly acute ischemic lesions and CMBs. These lesions were typically small, multifocal, and located in watershed or cortical regions. The findings suggest that MRI can reveal subclinical cerebral involvement and may serve as a useful tool for early diagnosis and risk assessment in IE [14]. 

Conventional angiography remains the gold standard for diagnosing intracranial mycotic aneurysms (ICMAs), given their frequent distal location within the cerebral arterial tree. While CTA and MRA reliably detect saccular aneurysms larger than 5 mm, their sensitivity is significantly lower for smaller lesions. One study reported sensitivities of 94% for CTA and 86% for MRA in detecting aneurysms ≥5 mm, but only 57% and 35%, respectively, for aneurysms <5 mm [20].

Microbiological diagnosis (blood culture and molecular methods)

Blood cultures constitute the primary diagnostic standard for microbiological confirmation of IE, with typical pathogens requiring two positive sets, while nontypical organisms require three or more. The update also includes PCR for *Coxiella burnetii*, *Bartonella* spp., and *Tropheryma whipplei*, and serologic tests, like high-titer IgG or immunofluorescence assays, enhancing diagnostic accuracy, particularly in culture-negative IE. These additions strengthen early detection and pathogen identification in atypical infections [3].

Blood biomarkers

Easily acquired blood biomarkers, indicative of the underlying biological processes (particularly inflammation or immune response), also have promising prospects as predictors. The presence of an EE in IE patients can be predicted by mean platelet volume, which is related to platelet function and activation [21].

A prospective study shows that baseline CRP level in the first three days of admission is a strong predictor of short-term adverse outcomes in IE patients [22,23].

Elevated plasma D-dimer levels at admission are also seen as an independent predictor of ischemic stroke in patients with IE [24,25]. Among 173 patients, those who developed stroke had significantly higher D-dimer levels [26,27], with a threshold ≥3393 μg/L showing good sensitivity (78%) and specificity (83%). *S. aureus* infection and mitral valve vegetations were also strongly associated with stroke risk [27,28].

Role of echocardiography in IERS

Transthoracic echocardiography (TTE) and TEE are the key diagnostic modalities for evaluation, diagnosis, and management of stroke in IE. In this regard, echocardiography is not only a powerful tool for the evaluation of cardioembolic sources of stroke, but also for establishing recommendations for the primary and secondary prevention of cardioembolic stroke [25]. 

**Figure 2:**
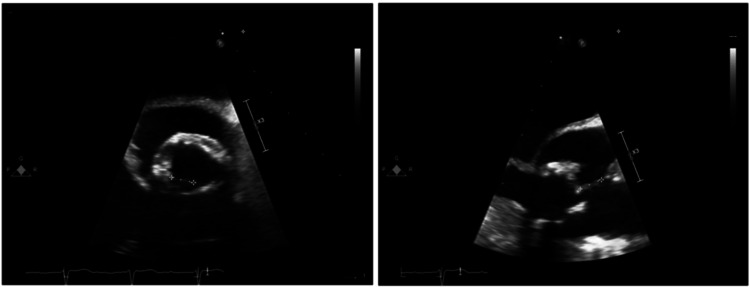
Transesophageal echocardiogram showing a mobile, echogenic vegetation attached to the non-coronary cusp of a prosthetic aortic valve Image credit: Puxeddu et al. [18] (CC BY 4.0)

TEE offers superior sensitivity (85%-90%) compared to TTE (Table 2), especially for detecting vegetations, abscesses, and perivalvular extensions [26]. As illustrated in Figure 2, TEE can detect vegetations on prosthetic valves, a critical finding in identifying cardioembolic sources of stroke in IE [18].

**Table 2:** Imaging modalities for detecting neurological complications in IE MRI, magnetic resonance imaging; DWI, diffusion-weighted imaging; TTE, transthoracic echocardiography; TEE, transesophageal echocardiography; CTA, computed tomography angiography; MRA, magnetic resonance angiography; DSA, digital subtraction angiography; IE, infective endocarditis

Modality	Utility/Detects	Performance/Strengths	Reference
Brain MRI (with DWI)	Detects acute ischemic lesions and cerebral microbleeds	Identifies silent lesions in a majority of asymptomatic cases	[14]
TTE	Initial screening for vegetations	Widely available, less sensitive than TEE	[26]
TEE	Vegetations, abscesses, peri-annular extension	High sensitivity (85%-90%) for detecting complications	[26]
CTA (head)	Intracranial aneurysms (≥5 mm)	Sensitivity ~94% for larger aneurysms; less accurate for <5 mm	[20]
MRA (head)	Intracranial aneurysms (≥5 mm)	Sensitivity ~86% for larger aneurysms; lower for <5 mm	[20]
DSA	Intracranial aneurysms (all sizes and distal lesions)	Gold standard, highly sensitive but invasive	[20]

General medical management of IE with stroke risk

The risk of stroke in IE decreases after commencement of antimicrobial therapy. A prospective cohort study of 1,437 patients demonstrated that the incidence of stroke among those receiving appropriate antibacterial therapy was 4.82 per 1,000 patient-days during the first week of treatment, decreasing to 1.71 per 1,000 patient-days in the second week, with a continued decline thereafter [27].

The American Heart Association (AHA) underscores the importance of prompt initiation of intravenous antibiotics in patients with IE, particularly those at risk of stroke. Empiric therapy should begin immediately after obtaining multiple blood cultures, as early antimicrobial intervention reduces the risk of septic embolism [28]. The 2020 ACC (American College of Cardiology)/AHA guideline reinforces the need for immediate empiric intravenous antibiotic therapy in suspected IE cases following blood cultures [29].

ATT in IE with stroke

Initiating antiplatelet or anticoagulant therapy solely for stroke prevention is not recommended in patients with newly diagnosed IE. In cases of IE with stroke or suspected stroke, these therapies should generally be temporarily discontinued, especially if high-risk features are present, e.g., large or mobile vegetations, *S. aureus* or *Candida* infections. If patients have a clear preexisting indication for ATT and are not surgical candidates, continuation may be considered if stroke is excluded and risk factors are absent. When surgery is planned, a short-acting anticoagulant (e.g., IV or low-molecular-weight heparin) may be used as a bridging strategy [30,31]. 

The AHA also strongly advises against initiating anticoagulation or antiplatelet therapy solely for stroke prevention in IE [32-34].

There is some disparity seen in the ATT recommendations. A meta-analysis of 12 studies involving 12,151 patients concluded that ATT in IE patients was not associated with a higher frequency of cerebrovascular events or ICH. Moreover, they found that the use of anticoagulation was associated with decreased in-hospital mortality, and the use of antiplatelets was associated with decreased systemic thromboembolism [31].

The management of comorbid conditions, including heart failure and sepsis, remains central to improving patient outcomes. Heart failure due to valvular destruction is a frequent and deadly complication, requiring early surgical consultation. Multidisciplinary care and individualized decision-making are emphasized throughout the management of IE [33,34].

Reperfusion therapy in IE-associated ischemic stroke

According to ESC 2023 Guidelines, mechanical thrombectomy (MT) can be performed in patients with IE-related large vessel occlusion (LVO) stroke after multidisciplinary discussion. It is not contraindicated, but should be weighed against hemorrhagic risks and vessel fragility [32]. According to a case report, MT appears to be a safe and effective therapeutic option in IE-related AIS with proximal-artery occlusion [33].

A multicenter study of consecutive IE cases treated with thrombectomy at nine stroke centers in Spain from 2011 to 2022 compared the efficacy and safety of MT in patients with IERS versus non-IE stroke cases. Among 50 matched IE cases and 200 controls, both groups had similar rates of successful recanalization (76% vs. 83%), neurological improvement at 24 hours, and favorable outcomes at three months. These findings suggest that thrombectomy is both effective and safe in IERS, and may be preferable to thrombolysis, supporting its inclusion in clinical guidelines [34].

In a systematic review of 37 articles involving 52 patients, intravenous thrombolysis in IERS was associated with a significantly higher risk of ICH - 4.14 times higher with thrombolysis alone, and 4.67 times higher with combined thrombolysis and thrombectomy. Although not statistically significant, there was a trend favoring thrombectomy in terms of better neurological improvement and independence [35].

In a large study, MT in IERS was evaluated in 34 cases. The data suggest that MT provides procedural success in restoring vessel patency. However, the functional benefit may be limited, likely due to underlying septic pathology, cerebral vessel fragility, and delayed diagnosis. Despite these limitations, MT remains a safer alternative to IV thrombolysis, which is contraindicated in IE because of the high hemorrhagic risk [36].

Timing of cardiac surgery after stroke in IE

Patients with IE are at increased risk for cardiovascular complications, like heart failure, uncontrolled infections, and EEs. Cardiac surgery is often needed to prevent these complications. Indications for cardiac surgery in these patients may include severe heart failure, severe valve dysfunction, prosthetic valve infection, invasion beyond the valve leaflets, recurrent systemic embolization, large mobile vegetations, or persistent sepsis despite adequate antibiotic therapy for more than five to seven days [37]. Timing of surgery becomes an important consideration when IE is complicated by stroke. It requires a multidisciplinary approach, with involvement of neurology, cardiology, and cardiothoracic surgery teams. Timing is decided by carefully evaluating the need for surgery against the risk of worsening stroke symptoms. The 2016 AATS (American Association for Thoracic Surgery) guidelines recommend a delay of one to two weeks for patients with non-hemorrhagic stroke, and three to four weeks for patients with hemorrhagic stroke or hemorrhagic conversion [37]. Surgery should not be performed in patients with severe neurological deficits until symptoms improve and the potential for recovery is evident [37]. In contrast, early surgery can be performed in patients with transient ischemic attack or silent cerebral embolism [38]. Assessing the risk of recurrent EEs and carefully evaluating the indications for valve replacement are critical steps in guiding optimal decision-making.

Several studies have evaluated the safety of cardiac surgery in patients with IE who present with neurologic deficits. Gillinov et al. [39] reported that most patients with ischemic stroke tolerated valve replacement well when surgery was delayed for approximately two to three weeks, with only 6% experiencing new or worsened neurologic deficits. Their data also suggest that patients with hemorrhagic stroke benefit from a longer interval before the operation, and they recommend delaying surgery for about four weeks in this group to minimize the risk of perioperative complications [39].

One meta-analysis showed that early surgery was associated with elevated perioperative mortality and higher neurological impairment, as compared to late surgery. The pooled analysis reported a relative risk of 1.74 for perioperative mortality (95% CI, 1.34-2.25; p < 0.0001; I² = 0%) and 2.09 for neurological impairment (95% CI, 1.32-3.32; p = 0.002; I² = 33%). Subgroup analyses indicated that, in ischemic stroke, early surgery performed within seven days carried comparable risks of perioperative mortality and neurological impairment to surgery within 14 days. In contrast, for hemorrhagic stroke, surgery undertaken before 21 days, as compared with before 28 days, showed trends toward higher perioperative mortality (RR 1.77 vs. 0.63; interaction p = 0.14) and neurological impairment (RR 2.02 vs. 0.44; interaction p = 0.11). No significant differences were observed in long-term mortality, although the available data were limited (Table 3) [40].

**Table 3:** Timing of cardiac surgery after stroke in IE IE, infective endocarditis; TIA, transient ischemic attack; peri-op, perioperative

Neurologic Status	Suggested Timing for Cardiac Surgery	Key Considerations	Reference
TIA or silent emboli	Early surgery reasonable	Prevents further emboli, low neurological risk	[38]
Ischemic stroke (non-hemorrhagic)	Delay 1-2 weeks; ≥11 days safer	Risk of hemorrhagic transformation peri-op	[37,39]
Hemorrhagic stroke or hemorrhagic conversion	Delay 3-4 weeks	High rebleed risk in the early period	[37,39]
Severe neurologic deficit, unclear recovery	Defer until improvement evident	Avoids futile surgery	[37]
Meta-analysis findings	Early surgery ↑ peri-op mortality and neurological complications	Benefit seen with delayed surgery, especially after a hemorrhagic stroke	[39]

Management of ICMAs

Mycotic aneurysms in IE are formed when friable cardiac vegetations produce septic emboli that stay in intracranial blood vessels at their branching points. These septic emboli can disrupt the blood flow in these vessels, leading to either cerebral infarction or promote infection. 

**Figure 3:**
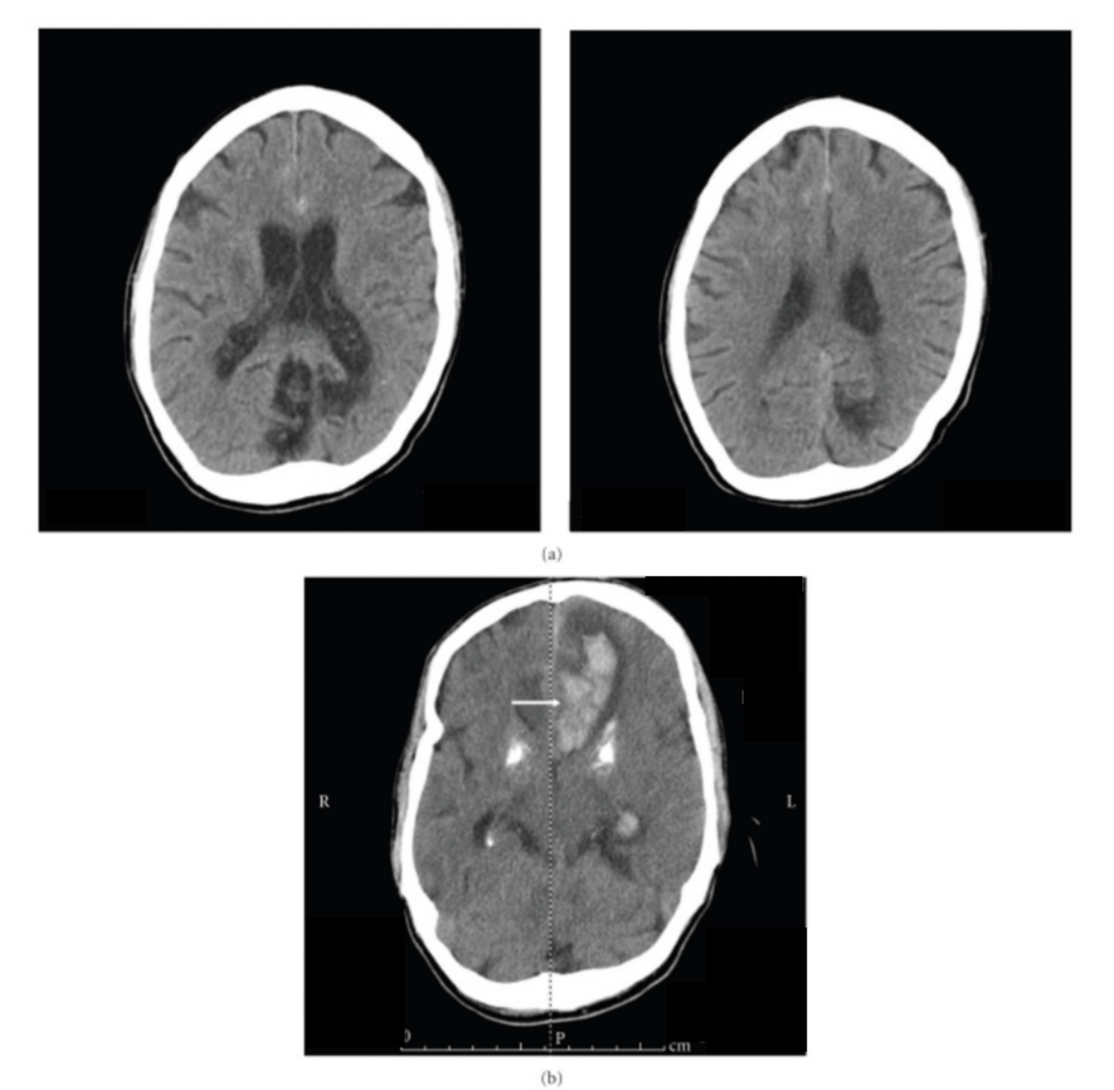
Noncontrast CT brain imaging (a) interhemispheric subarachnoid hemorrhage and (b) intraparenchymal hemorrhage 12 hours later, consistent with ruptured mycotic aneurysm (arrow). Image credit: Kuo et al. [41] (CC BY 4.0)

The vasa vasorum theory explains the pathogenesis in a very clear manner. This theory proposes that bacteria from septic emboli enter the vasa vasorum and cause severe inflammation in the adventitia layer. This inflammation penetrates further within the wall and makes it weak, leading to aneurysm formation. These aneurysms are more frequently present in the anterior circulation and carry a significant risk for hemorrhagic stroke [41]. Figure 3 illustrates the radiological progression of hemorrhagic stroke secondary to the rupture of a mycotic aneurysm, as demonstrated by Kuo et al. [41].

Imaging is crucial in the diagnosis and can be performed using CTA, MRA, or digital subtraction angiography (DSA). In the past, DSA was considered the gold standard, but nowadays, advancements in multi-detector CTA offer high-resolution imaging of intracranial blood vessels with low procedural risk. In a recent systematic meta-analysis, CTA had a sensitivity of 90% and a specificity of 86%. When compared to DSA, MRA is also an evolving imaging modality in the diagnosis of intracranial aneurysms. When compared to CTA, it is 87% sensitive and 95% specific; however, for aneurysms smaller than 3 mm, its sensitivity falls to 38%, versus 61% for CTA [41]. 

The actual prevalence of mycotic aneurysms in IE is still unknown; this could be because cerebral angiography is rarely done, and aneurysms may resolve with antibiotic treatment [42]. The clinical presentation of mycotic aneurysms includes fever, neurological symptoms, immunosuppression, vascular risk factors, and evidence of infection, including elevated C-reactive protein (CRP), erythrocyte sedimentation rate (ESR), leukocytosis, and positive blood cultures. Positive blood cultures are found in only 50%-85% of cases. Culture-negative IE is also reported in cases of cerebral mycotic aneurysm. Patients with risk factors and suggestive clinical findings should be evaluated for a mycotic aneurysm. Radiological findings are the most sensitive and specific indicators of a mycotic aneurysm [43].

Patients with left-sided and right-sided endocarditis, a patent foramen ovale (PFO), and ICH on CNS imaging were more likely to have mycotic aneurysms detected through DSA, as compared to patients without ICH. Routine DSA screening for mycotic aneurysms is usually not performed in patients with endocarditis undergoing evaluation for valve surgery who present without ICH on CNS imaging [44]. Management of intracranial infective aneurysms (IIAs) depends on several factors, including size, location, clinicians’ expertise, and rupture status. The most important factor in predicting prognosis is rupture. Ruptured aneurysms are associated with high mortality rates - up to 80% in one study - versus 30% mortality in those with unruptured aneurysms. The increased mortality could be related to rebleeding or to the initial hemorrhage itself [45].

Ruptured IIAs are often managed by either open or endovascular procedures, following a two- to three-week delay before cardiac valve replacement. Endovascular therapies are usually less invasive and are preferred in patients who are unfit for surgery due to cardiac disease [45]. There is controversy regarding the management of unruptured aneurysms. The available data are limited due to small sample sizes, lack of randomization, selection bias, and failure to include measures of morbidity in addition to mortality outcomes. A 2002 study reported on five patients who underwent endovascular repair for unruptured aneurysms and valve replacement within a week, without complication. A more recent review suggests that stable, small, unruptured aneurysms can be managed with antibiotics and serial imaging, while large, enlarging, or symptomatic unruptured aneurysms can be managed with antibiotics and endovascular treatment [45].

Controversies in ATT

There is uncertainty regarding the use of anticoagulants in IE patients with stroke. It is important to balance the benefits and the risks of bleeding after starting anticoagulants in these patients. IE is associated with vascular complications, such as cerebral embolism, ICH, and renal infarction. 

Although anticoagulation is the cornerstone treatment for thromboembolic complications, observational studies have shown that anticoagulation has failed to decrease the risk of ischemic stroke in IE patients. This suggests that IE alone is not an indication for the use of anticoagulation. Current recommendations are based solely on observational studies and expert opinions because of the lack of randomized controlled trials and high-quality meta-analyses. A multidisciplinary approach and patient engagement are required to determine the timing and regimen of anticoagulation in patients with IE, especially in specific situations - for example, receiving warfarin anticoagulation at the time of IE diagnosis, cerebral embolism or ischemic stroke, ICH, or urgent surgery [46].

IE patients are at high risk for both treatment-related and spontaneous bleeding. Intracerebral bleeding is the most severe complication in these settings and occurs in almost 5% of cases. Single antiplatelet therapy with low-dose aspirin after hospitalization for IE has been associated with decreased mortality within 90 days, without any increase in hemorrhagic stroke risk [47]. Embolization is another complication associated with IE. It is clinically apparent in up to 50% of IE patients and is known to be associated with *S. aureus *infection, large (>1 cm) vegetation, and mitral valve IE. Immediate initiation of antibiotic therapy has been the most effective strategy to decrease the rate of septic embolism. There is controversy regarding the use of anticoagulation therapy as a preventive strategy for EEs, particularly in patients already on anticoagulation, due to the increased risk of ICH. This increased risk could be related to large infarcts susceptible to hemorrhagic conversion, septic emboli causing arteritis and arterial rupture, or rupture of mycotic aneurysms from weakened vessel walls. Reports have shown higher rates of cerebral hemorrhage and mortality in anticoagulated IE patients [48], particularly those with *S. aureus* infection, which is a major reason to avoid ACT.

In contrast, some studies have shown that ACT was associated with a decreased risk of embolism without any increase in bleeding complications [48]. Another study evaluated the use of oral anticoagulant and antiplatelet agents in the development of embolic complications and bleeding episodes within 30 days of IE diagnosis. Of 129 patients with IE, 20 received anticoagulant/antiplatelet therapy, and 14 did not receive such therapy. The median age was 63 years, with 50% male patients. *S. aureus* was the infecting pathogen in 41% of patients. EEs occurred in 30% of patients receiving anticoagulant/antiplatelet therapy versus 7.1% of those not receiving therapy. No patients in the anticoagulant/antiplatelet therapy arm experienced a bleeding event, whereas 7.1% of those in the comparator arm did [49].

Interruption of antiplatelet and anticoagulant therapy is generally recommended if there is any intracranial or other major bleeding. In ischemic stroke, when there is no hemorrhage, oral anticoagulants should be replaced with unfractionated or low-molecular-weight heparin for 7-14 days, with close monitoring. In patients with intracranial bleeding and a mechanical valve, a multidisciplinary approach should be considered. In these patients, low-molecular-weight or unfractionated heparin should be resumed as soon as possible. In the absence of stroke, replacement of oral anticoagulant therapy with unfractionated or low-molecular-weight heparin for one to two weeks should be considered in cases of *S. aureus* infectious endocarditis, with close monitoring. Thrombolytic therapy is not advised in patients with IE [50]. 

## Conclusions

IE-related stroke poses complex diagnostic and therapeutic challenges that require multidisciplinary care, involving integration of cardiology, neurology, infectious disease, and surgical expertise. Advances in imaging, early antimicrobial therapy, timing of surgery, and careful use of reperfusion techniques are reshaping management. Ongoing research is needed to refine risk stratification and optimize individualized care.
